# Effect of Glutamate Concentration and Atmosphere of Incubation on the Production of ɣ-Aminobutyric Acid in *Levilactobacillus brevis* LB12

**DOI:** 10.3390/microorganisms14010108

**Published:** 2026-01-04

**Authors:** Emanuela Lavanga, Marilisa Giavalisco, Annamaria Ricciardi, Teresa Zotta

**Affiliations:** Department of Agricultural, Forestry, Food and Environmental Sciences, University of Basilicata, 85100 Potenza, Italy; emanuela.lavanga@unibas.it (E.L.); marilisa.giavalisco@unibas.it (M.G.); annamaria.ricciardi@unibas.it (A.R.)

**Keywords:** *Levilactobacillus brevis*, ɣ-aminobutyric acid, aerobiosis, *gad* operon

## Abstract

*Levilactobacillus brevis* is able to produce ɣ-aminobutyric acid (GABA), a non-proteogenic amino acid that provides several benefits to human health. In this study, we investigated the effect of glutamate (Glu) and oxygen (O_2_) on biomass yield, GABA production and regulation of the *gad* operon in *Lvb. brevis* LB12. A change in incubation atmosphere from anaerobiosis (AN) to aerobiosis (AE) was applied to elucidate if AE pre-adaptation and cultivation could be exploited to improve cell density, as well as to determine the role of O_2_ on the expression of the *gad* operon. AE increased biomass yield, but impaired Glu to GABA conversion, in both the cultivation and the adaptation phases. The *gad* operon (*gadR*, *gadC*, *gadB*, *gltX*) was up-regulated in the presence of Glu, while O_2_ strongly reduced the transcription of *gadC* and *gadB*. Switching the incubation atmosphere (AE vs. AN) and Glu supplementation did not restore the gene functionality, suggesting that the negative effect of O_2_ was persistent and more prolonged adaptation to AN would be required. This study provides additional data on the regulation of the *gad* operon, but further insight on the effect of O_2_ upon GABA production by *Lvb. brevis* must be expanded to understand the possible mechanisms involved.

## 1. Introduction

*Levilactobacillus brevis* is a heterofermentive lactic acid bacterium (LAB) used as starter and/or adjunct culture for the production of several fermented foods, especially vegetable- and cereal-based products [1,2]). Some *Lvb. brevis* strains, moreover, have been investigated for their potential probiotic features [3,4].

Most *Lvb. brevis* are able to synthetize ɣ-aminobutyric acid (GABA), a non-proteogenic amino acid that provides several benefits to human health [5,6,7]. In LAB, GABA production is regulated by the *gad* operon, which includes the genes encoding glutamate decarboxylase (*gadB*) and glutamate/GABA antiporter (*gadC*), responsible, respectively, for the irreversible decarboxylation of glutamate to GABA (GAD) and for the external secretion of GABA (GadC). The *gad* operon also includes the transcriptional regulator *gadR* (upstream of *gadC*), which positively regulates the expression of *gadB* and *gadC*, and a *gltX* sequence (glutamyl-tRNA synthase; in some LAB, it is located downstream of *gadB*), whose roles in GABA production need further investigation [8,9,10]. A stand-alone glutamate decarboxylase *gadA*, away from the *gad* operon, is also present in many LAB genomes, but it seems not to be directly involved in GABA synthesis [11].

Among LAB, most *Lvb. brevis* strains harbor the complete *gad* operon [12]. The presence of the *gad* operon is crucial for the production of GABA and studies on its regulation may be of practical relevance for boosting glutamate to GABA conversion in *Lvb. brevis*.

To date, many authors have investigated and characterized *Lvb. brevis* strains for their capability to produce GABA in synthetic and/or food matrices [13,14], highlighting that several factors, such as pH values, PLP cofactor, glutamate content, and genetic equipment, may affect the level of glutamate decarboxylation and GABA accumulation. Efficient conversion of glutamate to GABA certainly requires high cell density and, therefore, the optimal growth conditions of GABA producers should be satisfied.

LAB are generally recognized as O_2_-tolerant anaerobes that use fermentative pathways for the production of biomass and energy. Recently, several authors have demonstrated that the shift from anaerobic (AN) fermentative metabolism to aerobic (AE) and respiratory (RS) cultivation may result in several physiological advantages, including increase in biomass and robustness to oxidation, long-term starvation and freeze-drying stresses [15].

*Lvb. brevis* is an O_2_-tolerant species, and for many strains AE cultivation significantly improves cell density [16]; therefore, its aerobic phenotypes could be exploited as microbial factories for high-efficiency GABA production.

The effect of O_2_ on GABA production has recently been investigated in some *Lvb*. *brevis* [17,18], suggesting that AE cultivation impairs glutamate to GABA conversion compared to AN growth. Wu and Shah [17] demonstrated that AE conditions reduced both biomass and GABA production in *Lvb. brevis* NPS-QW 145 (an O_2_-sensitive phenotype), and acidification of the growth medium did not fully restore the GABA machinery in AE cells, although low pH generally promotes GAD activity; in *Lvb. brevis* NPS-QW 145 the transcription of the *gad* operon was also impaired in AE-growing cells. Zotta et al. [18] evaluated the effect of O_2_ in *Lvb. brevis* LB12 (an O_2_-tolerant strain), confirming that AE growth promoted biomass yield but reduced GABA accumulation in AE-growing cells.

In this study, we investigated the effect of glutamate induction and atmosphere of incubation (anaerobic vs. aerobic cultivation; AN vs. AE) on the biomass yield, GABA production and relative expression of the *gad* operon of *Lvb. brevis* LB12. We also evaluated whether the inhibitory effect of O_2_ on the GABA production pathway was irreversible by using a cultivation approach based on metabolic shift and AN or AE adaptation, in order to verify if AE cultivation could be exploited as a first step for the massive production of biomass, and the subsequent shift to AN conditions could be used to restore the Glu/GABA system and ensure a high conversion rate.

## 2. Materials and Methods

### 2.1. Strain and Culture Conditions

*Levilactobacillus brevis* LB12 (isolated from sourdough) was used in this study. The strain was maintained as freeze-dried stock (11% *w*/*v* skim milk with 0.1% *w*/*v* ascorbic acid) in the Unibas Yeasts and Bacteria Culture Collection (code UBYBCC015), Università degli Studi della Basilicata (Potenza, Italy), and routinely propagated in Weissella Medium Broth pH 6.8 (WMB [19]) before each assay.

*Lvb. brevis* LB12 was selected for its ability to produce ɣ-aminobutyric acid (GABA [18]), and here was used to evaluate the effect of glutamate induction and atmosphere of incubation (anaerobiosis vs. aerobiosis, i.e., AN vs. AE) on the biomass yield, GABA production, and expression of the *gad* operon genes. The optimal pH value and glutamate concentration (hereinafter Glu) to be used for LB12 cultivation and GABA production assays were preliminarily assessed as described in Section 2.2 and Section 2.3.

### 2.2. Assessment of Optimal pH for GABA Production in Buffer System

WMB precultures (24 h, 30 °C) of *Lvb. brevis* LB12 were standardized at a final absorbance at 650 nm (A_650_) of 1.0 (SmartSpec™ Plus Spectrophotometer, Bio-Rad Laboratories Inc., Milan, Italy) and used to inoculate (2% *v*/*v*) a modified WMB (mWMB; [18]) supplemented or not with monosodium glutamate (MSG 10 g/L; hereinafter mWMB+G and mWMB, respectively). At the end of incubation (40 h, 30 °C, static growth in screw cap bottles), the pH value (CyberScan-pH110meter, Oakton Instruments, Vernon Hills, IL, USA), cell density (A_650_) and biomass yield (cell dry weight, CDW, g/L) were measured. Supernatants were collected (8000 rpm, 5 min, 4 °C) and used to estimate the production of GABA with Thin Layer Chromatography (TLC [18]). Harvested cells were washed twice (8000 rpm, 5 min, 4 °C) with 20 mM potassium phosphate buffer pH 7 (PB7), standardized to a final biomass of 1 g/L and resuspended in a reaction buffer containing 50 mM sodium acetate, 0.1 mM pyridoxal-5′-phosphate (PLP) and 10 g/L of MSG [18], adjusted to a pH value of 3.5, 4.0, 4.5, 5.0, 5.5, or 6.0. Reaction mixtures were incubated for 4 h at 37 °C and 150 rpm (orbital shaker, Heidolph Unimax 2010, Heidolph Instruments GmbH & Co. KG, Nuremberg, Germany) as reported in Zotta et al. [18]. At the end of incubation, the pH value, cell density (A_650_), and GABA accumulation (TLC) were measured. Two biological replicates were carried out.

### 2.3. Assessment of Optimal Glutamate Concentration for GABA Production in Synthetic Medium

WMB precultures (cultivated at 30 °C for 24 h; standardized at A_650_ of 1.0) were used to inoculate (2% *v*/*v*) mWMB supplemented with different glutamate (Glu) concentrations (0, 1, 2, 5 or 10 g/L of MSG, corresponding to 0, 5.9, 11.8, 29.6 or 59.1 mM of Glu). At the end of incubation (40 h, 30 °C, static condition), the pH value, cell density (A_650_), biomass production and GABA accumulation in growth medium (TLC assay) were measured. Cells collected (8000 rpm, 5 min, 4 °C) from the different Glu-supplemented mWMB were standardized (1 g/L) and incubated in the reaction buffer at pH 4.5 (optimum pH resulting from the previous assay, Section 2.2) at 37 °C, 4 h, 150 rpm, as described before. At the end of incubation, the pH value, cell density (A_650_), and GABA accumulation (TLC) were measured. Two biological replicates were carried out.

### 2.4. Effect of Glutamate Induction and Atmosphere of Incubation on Biomass Yield and GABA Production

**Figure 1:**
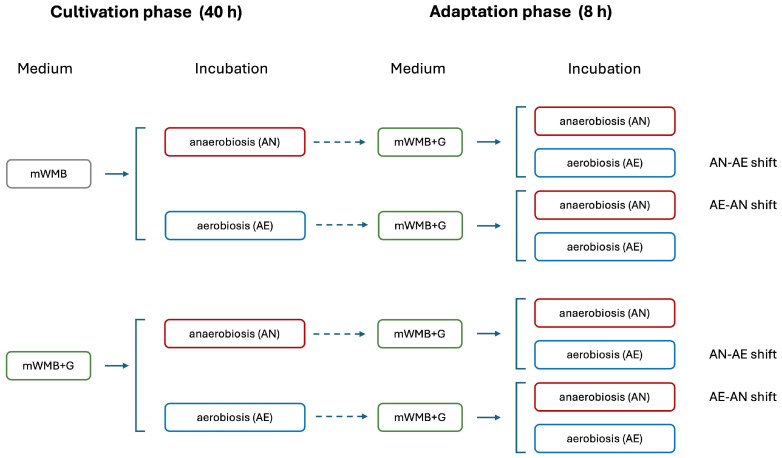
Substrates and incubation atmospheres used for cultivation (40 h) and adaptation (8 h) phases (as reported in Section 2.4).

Standardized WMB precultures (24 h, 30 °C; A_650_ of 1.0) were inoculated (2% *v*/*v*) in mWMB or mWMB+G (10 g/L MSG, the optimal concentration resulting from previous assay; Section 2.3) and incubated in anaerobic (AN; static growth in screw-cap bottles) and aerobic (AE; shaken flasks, 150 rpm) conditions, at 30 °C for 40 h [18]. At the end of incubation, the pH value, cell density (A_650_), biomass yield and GABA accumulation in growth medium (TLC assay) were measured. Stationary cells collected (8000 rpm, 5 min, 4 °C) from each growth condition (mWMB-AN, mWMB-AE, mWMB+G-AN, mWMB+G-AE) were washed twice in PB7, resuspended in a new batch of mWMB+G (maintaining the same volume used for the previous cultivation), and incubated in both AN and AE conditions for 8 h at 30 °C to reach a metabolic shift and adaptation phase, as reported in Figure 1.

During the adaptation phase, samples were aseptically withdrawn at 2 h intervals for detecting pH, cell density (A_650_), biomass yield and GABA accumulation (TLC) in both the growth medium and the buffer system. After 4 h or 8 h of adaptation, the relative expression of genes belonging to the *gad* operon (see Section 2.5) was evaluated as described below.

### 2.5. Effect of Glutamate Induction and Atmosphere of Incubation on Relative Expression of GAD Operon Genes

#### 2.5.1. In Silico Analysis and Primer Design

Sequences of genes belonging to the *gad* operon (i.e., *gadB*/*gadA*, glutamate decarboxylase; *gadC*, glutamate:gamma-aminobutyrate antiporter; *gadR*, transcriptional regulator; *gltX*, glutaminyl-tRNA synthetase or glutamate-tRNA ligase) were retrieved from 33 finished *Lvb. brevis* genomes, publicly available on the Integrated Microbial Genomes database (IMG, https://img.jgi.doe.gov/; on 1 April 2025).

For all genes, sequences were aligned and analyzed with MEGA software version 11.0.13 (ClustalW alignment), and the conserved regions were used to design primers (Table S1) with Primer Express software 4.1.0 [20] (University of Tartu, Tartu, Estonia; https://primer3.ut.ee).

#### 2.5.2. Extraction of Total RNA

Total RNA was isolated from cells collected after 40 h of cultivation in mWMB-AN, mWMB-AE, mWMB+G-AN and mWMB+G-AE (1st step in Figure 1), and from cells withdrawn after 4 h and 8 h for metabolic shift and adaptation phase (all conditions; second step in Figure 1). The ZR Fungal/Bacterial RNA kit (Zymo Research, Irvine, CA, USA) was used, and an on-column DNA-digestion step (DNase I RNase-free; Invitrogen™, Burlington, ON, Canada) was added to the RNA extraction protocol. RNA samples were quantified with a NanoDrop™ One/OneC Microvolume UV-VIS spectrophotometer (Thermo Scientific^TM^; Milano, Italy) and used as templates for the synthesis of complementary DNA (cDNA). One microgram of RNA was mixed with 4 μL of Maxima™ H Minus cDNA Synthesis Master Mix (Thermo Scientific^TM^, Waltham, MA, USA) and incubated as described in the manufacturer’s instructions. cDNA samples were stored at −80 °C before use.

#### 2.5.3. Quantitative RT-PCR and Gene Expression Analysis

qRT-PCR was performed in a StepOnePlus Real-Time PCR instrument (Applied Biosystems, Foster City, CA, USA), using a PowerTrack™ SYBR Green Master Mix (Applied Biosystems, Vilnius, Lithuania). The amplification program included 1 cycle at 95 °C for 2 min, 40 cycles at 95 °C for 5 s and 60 °C for 30 s, with a melting curve of 95 °C for 15 s, 60 °C for 1 min and 95 °C for 15 s (ramping rate 0.3 C/s). Reaction mixtures without a cDNA template were used as negative controls. Two technical replicates were carried out for each growth condition and biological replicate. The relative expression of all genes was estimated according to the ΔΔCt method [21], using glyceraldehyde-3-phosphate dehydrogenase (*gapdh*) as reference gene and AN cultivation in unsupplemented mWMB as reference growth condition.

### 2.6. Statistical Analyses

All statistics and graphs were created with R (version 4.5.0 [22]) and relevant R packages. Analysis of Variance (ANOVA) and post-hoc Tukey’s HSD (Honesty Significant Difference) test were used to estimate statistically significant differences (*p* ≤ 0.01).

Densitometric analysis of TLC spots was performed with NIS-Elements BR v5.41.00 (Nikon Instruments Inc., Tokyo, Japan), as described in Zotta et al. [18].

## 3. Results and Discussion

### 3.1. Assessment of Optimal pH and Glutamate Concentration for GABA Production by Lvb. brevis LB12

In this study, preliminary trials to identify the optimal pH and glutamate (Glu) concentration for GABA production by *Lvb. brevis* LB12 were carried out. The highest % of Glu uptake by cells (i.e., 50% of the total Glu concentration) and the highest % of Glu to GABA conversion (i.e., 95% of the available Glu) were measured at pH 4.5 (trial in buffer system; Figure S1). pH values lower or higher than 4.5 significantly impaired Glu consumption and GABA production, indicating that both glutamate decarboxylase (GAD) and Glu/GABA antiporter (GadC) were affected by pH.

As expected, the Glu intake and, consequently, the efficiency of GABA synthesis proportionally increased with Glu concentration (trial in growth medium; Figure S2), and the highest values were measured in LB12 cells cultivated in mWMB supplemented with 10 g/L MSG (i.e., 59.1 mM Glu). A very low amount of GABA was also found in unsupplemented mWMB, as a low content of glutamate (i.e., 7.6 mM) was present in the cultivation medium. The small amount of Glu naturally present in mWMB was considered in the calculations of GABA production and % efficiency for all trials.

Liu et al. [23] tested different pH values (4.0, 4.5, 5.0, 5.5, 6.0) and Glu concentrations (0, 0.25, 0.50, 0.75, 1.00, 1.25, 1.50, 2.00% *w*/*v*) in *Lvb. brevis* YSJ3, confirming that GABA biosynthesis was higher in the presence of 1.25% Glu and pH 4.5. Previously, Banerjee et al. [9] demonstrated that *Lvb. brevis* Lbr-610 was able to consume a high amount of Glu (90 g/L) during prolonged cultivation, but the % of Glu/GABA conversion was not strongly correlated to Glu content, as other factors, such as the increase in pH in the growth medium, could occur and affect the Glu/GABA system. These data suggest that optimal pH and Glu concentrations are strain-specific and need to be optimized on the basis of cultivation and GABA production processes.

### 3.2. Effect of Glutamate Induction and Atmosphere of Incubation on Biomass Yield and GABA Production by Lvb. brevis LB12

**Figure 2:**
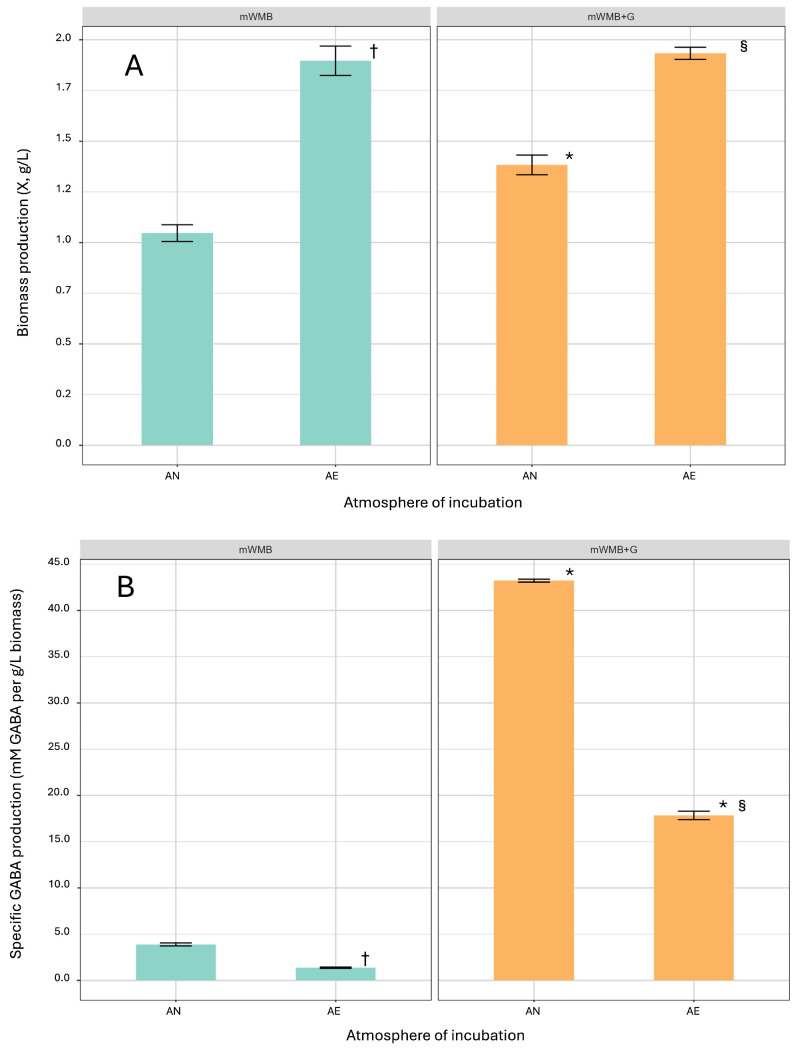
(**A**) Biomass production (X, g/L; *Y*-axis) and (**B**) specific GABA production (GABA/X; GABA out, mM/produced biomass, g/L) by *Levilactobacillus brevis* LB12 cultivated (40 h) in mWMB supplemented (mWMB+G; orange bars) or not (mWMB; green bars) with monosodium glutamate (MSG 10 g/L, i.e., 59.1 mM glutamate), under anaerobic (AN) and aerobic (AE) conditions (*X*-axis). GABA was measured in substrate supernatants. * significant differences (*p* < 0.01, Tukey’s HSD multiple comparisons) between unsupplemented and supplemented mWMB under the same growth condition (AN or AE); ^†^ significant differences (*p* < 0.01) between AN and AE cultivation in unsupplemented mWMB; ^§^ significant differences (*p* < 0.01) between AN and AE cultivation in supplemented mWMB+G.

The effect of Glu (10 g/L MSG, i.e., 59.1 mM Glu) and the incubation atmosphere (anaerobiosis vs. aerobiosis, i.e., AN vs. AE) on biomass production and GABA accumulation was evaluated in *Lvb. brevis* LB12 after 40 h of cultivation in Glu-supplemented and unsupplemented WMB (Figure 2).

As expected (an O_2_-tolerant phenotype was previously observed in *Lvb. brevis* LB12; [16,18]), AE cultivation significantly increased the biomass yield of LB12, regardless of Glu supplementation (Figure 2A), but strongly impaired the specific GABA production (Figure 2B).

**Figure 3:**
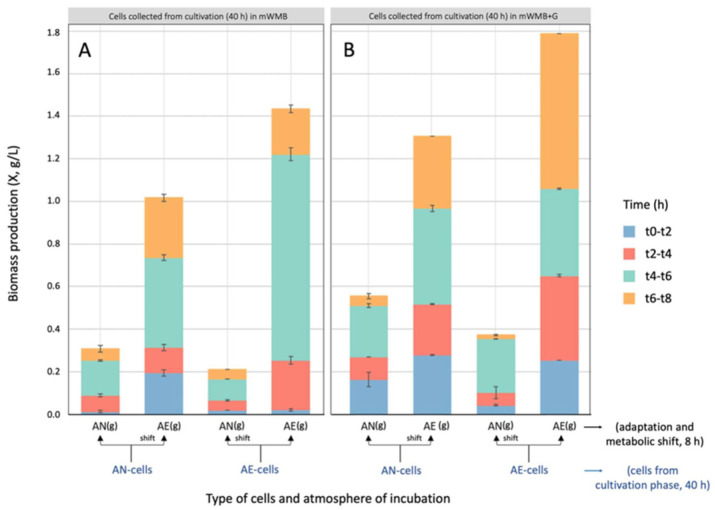
Biomass production (X, g/L; *Y*-axis) by *Levilactobacillus brevis* LB12 during the adaptation phase (8 h of cultivation; metabolic shift) to anaerobic (AN) and aerobic (AE) conditions in supplemented mWMB+G (with MSG 10 g/L, i.e., 59.1 mM glutamate). *X*-axis: adapted and metabolically shifted cells previously cultivated (40 h) in AN or AE state (AN-cells vs. AE-cells) in unsupplemented (mWMB; panel (**A**)) or Glu-supplemented (mWMB+G; panel (**B**)) medium. Colour in stacked bars (adaptation phase): blue, t0–t2, biomass production from 0 to 2 h of incubation; red, t2–t4, biomass production from 2 to 4 h of incubation; green, t4–t6, biomass production from 4 to 6 h of incubation; orange, t6–t8, biomass production from 6 to 8 h of incubation. Total biomass production was significantly different (Tukey’s HSD, *p* ≤ 0.01) in all conditions.

Data collected during the adaptation phase (Figure 3) confirmed that maintenance of the AE state (cultivation in AE and subsequent adaptation to AE) and the shift from AN to AE conditions (cultivation in AN and subsequent adaptation to AE) significantly increased biomass yield, suggesting that AE growth is a convenient strategy to boost cell density in *Lvb. brevis* LB12. Prolonged adaptation (up to 8 h) to AE and Glu supplementation further increased biomass production.

**Figure 4:**
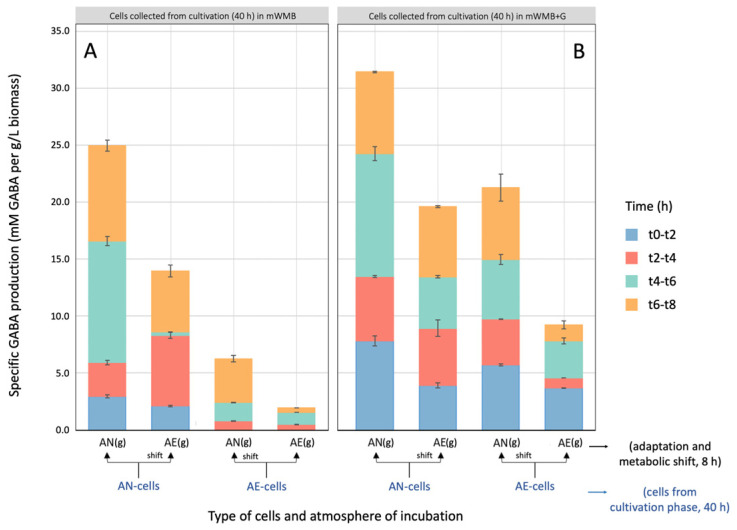
Specific production of GABA (GABA/X; GABA out, mM/produced biomass, g/L) by *Levilactobacillus brevis* LB12 during the adaptation phase (8 h of cultivation; metabolic shift) to anaerobic (AN) and aerobic (AE) conditions in supplemented mWMB+G (with MSG 10 g/L, i.e., 59.1 mM glutamate). GABA was measured in substrate supernatants. *X*-axis: adapted and metabolically shifted cells previously cultivated (40 h) in AN or AE state (AN-cells vs. AE-cells) in unsupplemented (mWMB; panel (**A**)) or Glu-supplemented (mWMB+G; panel (**B**)) medium. Colour in stacked bars (adaptation phase): blue, t0–t2, biomass production from 0 to 2 h of incubation; red, t2–t4, biomass production from 2 to 4 h of incubation; green, t4–t6, biomass production from 4 to 6 h of incubation; orange, t6–t8, biomass production from 6 to 8 h of incubation. Total GABA concentration was significantly different (Tukey’s HSD, *p* ≤ 0.01) in all conditions.

**Figure 5:**
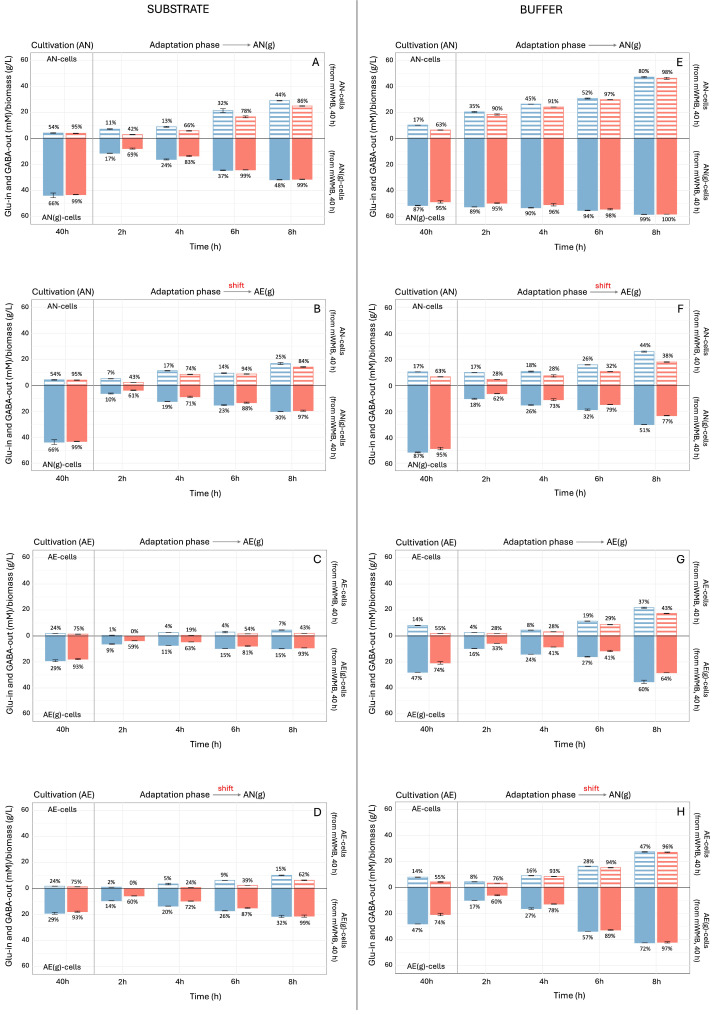
Glutamate uptake (Glu-in; blue bars) and GABA production (GABA-out; red bars) (Glu or GABA, mM/biomass, g/L) by *Levilactobacillus brevis* LB12, during cultivation (40 h) and adaptation phase (8 h of incubation). Glu in and GABA out were measured in both substrate supernatants (panels (**A**–**D**)) and buffer system (panels (**E**–**H)**). Cultivation section: growth (40 h) in mWMB supplemented (mWMB+G; filled bars) or not (mWMB; dashed bars) with monosodium glutamate (MSG 10 g/L, i.e., 59.1 mM glutamate), under anaerobic (AN) and aerobic (AE) conditions. Adaptation phase: 8 h of cultivation in supplemented mWMB+G (with MSG 10 g/L, i.e., 59.1 mM glutamate), with a metabolic shift of cells previously cultivated (40 h) in the AN or AE state (AN-cells vs. AE-cells; AE-cells vs. AN-cells). In the adaptation phase, Glu-in and GABA-out were measured at 2 h intervals. % on bars: blue, efficiency of Glu uptake; red, efficiency of Glu/GABA conversion.

However, despite the high cell density, the shift to AE conditions significantly reduced the specific GABA production compared to the levels measured in AN-growing and AN-adapted cells (Figure 4). Specifically, the presence of O_2_ impaired both Glu intake (% on blue bars) and the efficiency (% on red bars) of Glu/GABA conversion (Figure 5, panels A–D), suggesting an inhibitory effect on both GAD and Glu/GABA antiporter. However, the major effect of atmosphere of incubation observed, in both the cultivation (40 h) and adaptation (8 h) phases, was that on the % of Glu uptake rather than on the % of Glu/GABA transformation, suggesting that O_2_ mainly impaired the activity of Glu/GABA antiporter rather than GAD. Our data confirmed the inductive effect of Glu (Figure 4 and Figure 5), because within the same incubation atmosphere between the cultivation and adaptation phases, cells previously grown in the presence of Glu (40 h, mWMB+G) had a greater capability to produce GABA. The % of Glu uptake and Glu/GABA conversion measured in the buffer system (Figure 5, panels E–H) confirmed the higher efficiency of the Glu/GABA pathway in AN-growing and AN-adapted cells, and the reduced activity when AE cultivation was applied.

### 3.3. Effect of Glutamate Induction and Atmosphere of Incubation on the Relative Expression of Gad Operon in Lvb. brevis LB12

**Figure 6:**
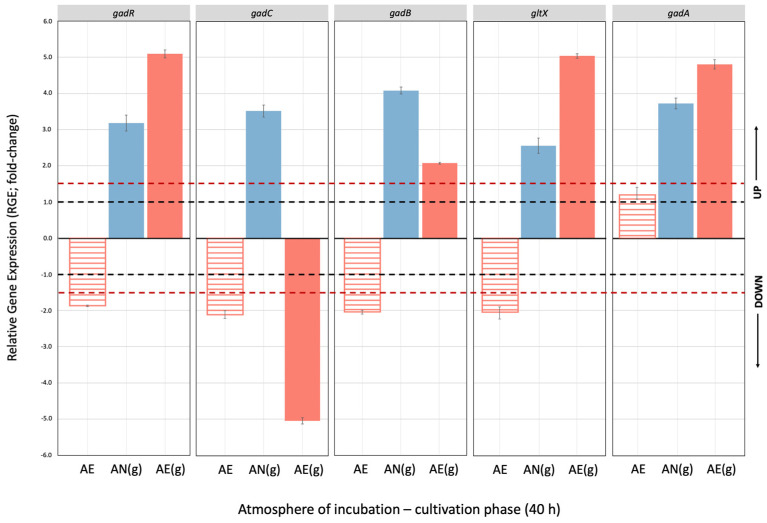
Relative gene expression (RGE) of *gadR* (transcriptional regulator), *gadC* (glutamate:gamma-aminobutyrate antiporter), *gadB*/*gadA* (glutamate decarboxylase) and *gltX* (glutaminyl-tRNA synthetase) genes of *Levilactobacillus brevis* LB12 cultivated (40 h) in unsupplemented (dashed bars) and Glu-supplemented (filled bars) mWMB medium, under anaerobic (AN) and aerobic (AE) conditions; (g) indicates mWMB supplementation with 10 g/L MSG (i.e., 59.1 mM glutamate). Mean values of three biological and two technical replicates are shown. UP, up-regulation (positive fold change); DOWN, down-regulation (negative fold change). Values ≥ and ≤ than +/−1.5-fold change (red dotted lines) indicate significant differences (Tukey’s HSD, *p* ≤ 0.01) in RGE compared to the reference growth conditions (AN, unsupplemented mWMB; black dotted lines).

The relative gene expression (RGE) of genes belonging to the *gad* operon (i.e., *gadR*, *gadC*, *gadB*, *gltX*) in response to Glu supplementation and atmosphere of incubation, after 40 h of incubation (stationary phase cells), is reported in Figure 6. Unsupplemented AN cultivation was used as reference condition (dotted black lines), while RGE values ≥ or ≤ than a +/−1.5-fold change (red dotted lines) indicated significant differences (Tukey’s HSD, *p* ≤ 0.01) in gene transcription.

**Figure 7:**
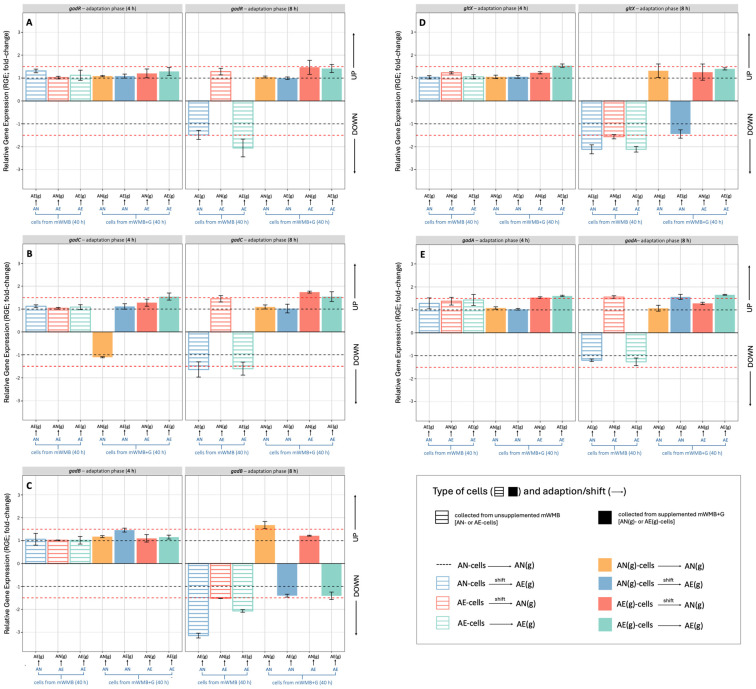
Relative gene expression (RGE) of *gadR* (transcriptional regulator, panel (**A**)), *gadC* (glutamate:gamma-aminobutyrate antiporter, panel (**B**)), *gadB*/*gadA* (glutamate decarboxylase, panel (**C**) and (**E**)) and *gltX* (glutaminyl-tRNA synthetase, panel (**D**)) genes of *Levilactobacillus brevis* LB12 during the adaptation phase (4 and 8 h incubation) to anaerobic [AN(g)] and aerobic [AE(g)] conditions. Adapted and metabolically shifted cells were previously cultivated (40 h) in unsupplemented (dashed bars) and Glu-supplemented (filled bars) mWMB medium, under anaerobic (AN) and aerobic (AE) conditions; (g) indicates mWMB supplementation with 10 g/L MSG (i.e., 59.1 mM glutamate). Mean values of three biological and two technical replicates are shown. UP, up-regulation (positive fold change); DOWN, down-regulation (negative fold change). Values ≥ and ≤ than +/−1.5-fold change (red dotted lines) indicate significant differences (Tukey’s HSD, *p* ≤ 0.01) in RGE compared to the reference growth conditions (AN, unsupplemented mWMB; black dotted lines).

As expected, the presence of Glu in AN-growing cells (blue bars) significantly promoted the transcription of all *gad* operon genes, but strongly impaired the relative expression of *gadC* in AE cells (−5.0-fold change), confirming that Glu/GABA antiporter was strongly affected by O_2_ and the presence of Glu was not able to restore its functionality. Contrarily, the presence of Glu induced transcription of *gadR* and *gltX* also in AE-growing cells, demonstrating that the expression of these genes was mainly related to Glu addition rather than incubation atmosphere. The presence of O_2_ impaired the expression of all *gad* operon genes in non-supplemented AE cells, but did not affect *gadA*, suggesting that the latter gene had different regulation than those of the *gad* operon. The switch of incubation atmosphere and the prolonged adaptation to AN and Glu-supplemented conditions (Figure 7; 8 h) did not restore the expression of *gad* operon genes, indicating that LB12 cells need additional time to resume gene transcription. For cells cultivated in unsupplemented mWMB, the shift to the AE state significantly impaired the expression of *gad* operon genes, despite Glu addition.

Several authors [10,12] demonstrated the presence of the complete *gad* operon in *Lvb. brevis*. Our data confirmed the co-regulation of *gadR*, *gadC*, *gadB*, and *gltX* in response to Glu supplementation, as already reported [9,11], but highlighted a different behavior of *gadC* under AE conditions suggesting that the transcription profile of the *gad* operon may differ depending on the O_2_ availability. Regulation of *gad* operon genes has been extensively investigated in response to Glu addition and pH values (generally demonstrating a positive correlation between acid stress robustness and *gad* expression [8,11]), but limited data are available on the effect of O_2_.

Wu and Shah [17] first demonstrated that AE conditions reduced growth, GABA production and the expression of *gad* operon in *Lvb. brevis* NPS-QW 145. Specifically, RGE of *gadR* (+10-fold change), *gadC* (+60-fold change) and *gadB* (+45-fold change; in Wu and Shah [17] annotated as *gadA*) was significantly higher in AN-growing cells compared to AE ones. The behavior of the *gad* operon in *Lvb. brevis* NPS-QW 145 [17] and *Lvb. brevis* LB12 (this study) was comparable as, in both strains, *gadB* and *gadC* were mostly affected by O_2_. However, strain NPS-QW 145 is an O_2_-sensitive phenotype (as indicated by viable count in AE and AN conditions) and, therefore, AE cultivation probably impaired the strain performance in a more complex way. The acidification of growth medium to pH 5 only partially restored GABA production in aerated cells of *Lvb. brevis* NPS-QW 145, confirming the strong inhibitory effect of O_2_. Subsequently, Zotta et al. [18] evaluated the effect of AE cultivation on the O_2_-tolerant strain *Lvb. brevis* LB12 (also used in this study), confirming that AE growth promoted biomass yield but impaired GABA accumulation in AE-growing cells. More recently, Ding et al. [24] also demonstrated that AE conditions, although increasing cell density, significantly decreased GABA production and *gad* operon expression in *Lvb. brevis* CGMCC 24975. Specifically, *gadR* (+30.54-fold change), *gadC* (+83.76-fold change) and *gadB* (+88.41-fold change) were strongly up-regulated in AN conditions. The stand-alone gene *gadA* was not affected by atmosphere of incubation. Both Wu and Shah [17] and Ding et al. [24] did not investigated the effect of O_2_ on the *gltX* gene.

Based on the above considerations, in this study we applied changes in incubation atmosphere (AN vs. AE; AE vs. AN) as a strategy to investigate the effect of O_2_ on the Glu/GABA conversion and regulation of *gad* operon genes, and to evaluate whether a switch to AN conditions could restore the functionality of the GABA system in AE-growing cells. Unfortunately, the shift and adaptation towards the AN state did not resume the activation of the Glu/GABA pathway, suggesting that the negative effect of O_2_ was severe. This study, moreover, demonstrated that the factors promoting the growth of *Lvb. brevis* (such as the AE conditions) does not always correspond to the optimal parameters for GABA production. The expression profile of the *gad* operon of *Lvb. brevis* LB12 (Figure 7, this study), however, showed that AE growth (in presence of glutamate) had a negative effect mainly on *gadC* (Glu/GABA antiporter; probably because it impairs the proton motive force across the membrane), while the other genes were weakly (*gadB*) or not at all affected (*gadR*, *gltX*) by O_2_, suggesting that the atmosphere of incubation impacts the regulation of the *gad* operon in a more complex way and further studies are needed to elucidate the role of O_2_ in order to optimize the cultivation and GABA production processes of *Lvb. brevis*.

Regulation of the *gad* operon, however, is strain-dependent and may be affected by other factors. Banerjee et al. [9] found different gene expression in some *Lvb. brevis* strains also in response to incubation time and growth phase. *Lvb. brevis* Lbr-6108 exhibited GABA production already during the early growth stage (6 h), consistent with early activation of the *gad* operon, while strains Lbr-35 and ATCC 14,869 accumulated significant amounts of GABA only after 24–48 h of incubation. In *Lvb. brevis* YSJ3 [23] the GABA production rate was higher from 8 to 30 h of incubation, while early (0–7 h) or late (>31 h) growth phases significantly impaired the GABA system, as also indicated by the low expression of *gad* operon genes (*gadR*, *gadC*, *gadB*).

In our study, *Lvb. brevis* LB12 accumulated GABA during prolonged cultivation (40 h), while the time of adaptation to the metabolic shift (up to 8 h) was not sufficient to induce significant transcription of *gad* operon genes and to restore GABA production, suggesting that in *Lvb. brevis* LB12 the incubation time and growth phase were also critical factors for the activation of the GAD system, and tailored cultivation processes should be optimized. Our data confirmed that *gadA* was not directly correlated to GABA production (as already demonstrated [9,23,25]), but could be involved in other glutamate-related mechanisms, and its role needs further investigation. The *gltX* gene, which catalyzes the attachment of glutamate to the corresponding tRNA, has been identified as part of the *gad* operon in *Lvb. brevis* by several authors ([9,10,13]; mainly through genome and sequence occurrence analyses), but its role in GABA production was poorly elucidated. We found an expression profile similar to *gadR* in all conditions, confirming a co-transcriptional mechanism, but partially similar to *gadB* and *gadC* only for the AN condition (while the behavior in presence of O_2_ was different), suggesting that regulation of the *gad* operon could be more complex. Banerjee et al. [9] demonstrated a full co-transcription for *gadR*, *gadC*, *gadB* and *gltX* in *Lvb. brevis* Lbr-6108, while Cataldo et al. [25] highlighted the presence of two transcriptional units (*gadR* and *gadC* units; *gadC*, *gadB* and *gltX* units) in *Lvb. brevis* CRL2013, confirming that the activation and regulation of the *gad* operon could be strain-specific and differ in response to several factors.

## 4. Conclusions

In this study, we evaluated the effect of Glu induction and atmosphere of incubation (AN vs. AE) on the GABA production and regulation of *gad* operon genes in *Lvb. brevis* LB12. Indeed, although the ability to synthesize GABA is widely distributed in the species *Lvb. brevis*, several factors may affect the efficiency of Glu/GABA conversion, resulting in low GABA yield. Although the detrimental effect of O_2_ on GABA production by *Lvb. brevis* LB12 was already observed (our previous data; [18]), this study provided further insight on the relative expression of the *gad* operon in response to AE cultivation; specifically, we demonstrated that the behavior of *gadR*, *gadC*, *gadB* and *gltX*, although belonging to the same gene cluster, may differ in response to O_2_ and interaction between AE growth and Glu supplementation.

Many members of *Lvb. brevis*, in fact, have an O_2_-tolerant phenotype, and the shift towards AE conditions may be exploited as a useful strategy to improve cell density and stress robustness, in order to develop more competitive starter and/or functional cultures. For *Lvb. brevis* LB12, however, AE growth and a following switch to the AN condition was not an adequate strategy for GABA production because, despite a greater biomass yield reached in the AE state, the Glu/GABA system was strongly compromised. Therefore, further studies (e.g., membrane-associated mechanisms) could be useful to elucidate the effect of O_2_ in the regulation of *gad* operon genes. Moreover, controlled cultivations (e.g., batch, fed-batch in bioreactor) modulating other parameters (e.g., pH values, incubation time, growth phase) could be exploited to implement a suitable process for concurrent production of high-yield biomass, robust cells, and GABA accumulation.

## Data Availability

The original contributions presented in this study are included in the article/Supplementary Materials. Further inquiries can be directed to the corresponding author.

## Supplementary Materials

The following supporting information can be downloaded at: https://www.mdpi.com/article/10.3390/microorganisms14010108/s1, Figure S1: Production of GABA from *Levilactobacillus brevis* LB12 in the reaction buffer (Section 2.2); Figure S2: Production of GABA from *Levilactobacillus brevis* LB12 cultivated in mWMB supplemented with different glutamate concentrations (Section 2.3); Table S1: Forward (F) and reverse (R) primer sequences used for the quantification of relative gene expression in *Lvb. brevis* LB12.

## Abbreviations

The following abbreviations are used in this manuscript:

GABA	ɣ-aminobutyric acid
TLC	Thin Layer Chromatography
*Lvb*	*Levilactobacillus*
LAB	Lactic Acid Bacteria
