# Supplementary material for: Effect of Glutamate Concentration and Atmosphere of Incubation on the Production of ɣ-Aminobutyric Acid in Levilactobacillus brevis LB12
Source: Microorganisms. 2026 Jan 4;14(1):108. doi: 10.3390/microorganisms14010108 (PMC12843658; doi:10.3390/microorganisms14010108)
Supplement: Supplementary file 1 [file microorganisms-14-00108-s001.zip › microorganisms-4051056-supplementary.pdf]

Effect of glutamate concentration and atmosphere of incubation on the production of ɣ-aminobutyric acid in *Levilactobacillus brevis* LB12

Emanuela Lavanga [1], Marilisa Giavalisco [1], Annamaria Ricciardi [1] and Teresa Zotta [1,*]

[1] Department of Agricultural, Forestry, Food and Environmental Sciences, University of Basilicata, 85100 Potenza, Italy; emanuela.lavanga@unibas.it (E.L); marilisa.giavalisco@unibas.it (M.G.); annamaria.ricciardi@unibas.it (A.R.); teresa.zotta@unibas.it (T.Z.). * Correspondence: teresa.zotta@unibas.it; Tel +39-0971-205563

Table S1. Forward (F) and reverse (R) primer sequences used for the quantification of relative gene expression in *Lvb. brevis* LB12.

Gene [a]	Primer	Sequence (5'–3')	Amplicon length [b]
gadA	gadA-F	5’-TCTCCCACAGTGCCTCCCAA-3’	175 bp
	gadA-R	5’-ATCAGCGGTAACTCGTGGCC-3’	
gadB	gadB-F	5’-CGGATTCACGTTGATGCTGCCT-3’	150 bp
	gadB-R	5’-AACAATCCACCCCAACCCAGG-3’	
gadC	gadC-F	5’-TGCGCATCAGAACTTTTGTCA-3’	193 bp
	gadC-R	5’-GCGAACAATCCCCTGTAAGCC-3’	
gadR	gadR-F	5’-TGCGCATCAGAACTTTTGTCA-3’	162 bp
	gadR-R	5’-AGTAATAGACCCGATTCATTGCG-3’	
gltX	gltX-F	5’-GCGGCACTATCACGGAACCA-3’	154 bp
	gltX-R	5’-CGGTGCGTACTTGGGATTGG-3’	
gadph	gadph-F	5’-TCCATGCCTTCACTGCTACGC-3’	171 bp
	gadph-R	5’-CCCGTTGTGCATGGCCTTGT-3’	

[a] *gadA* and *gadB*: glutamate decarboxylase; *gadC*: glutamate:γ-aminobutyrate antiporter; *gadR*: transcriptional regulator; *gltX*: glutamyl-tRNA synthetase; *gapdh*: glyceraldehyde-3-phosphate dehydrogenase, used as the reference gene. [b] Amplicon length in base pairs (bp).

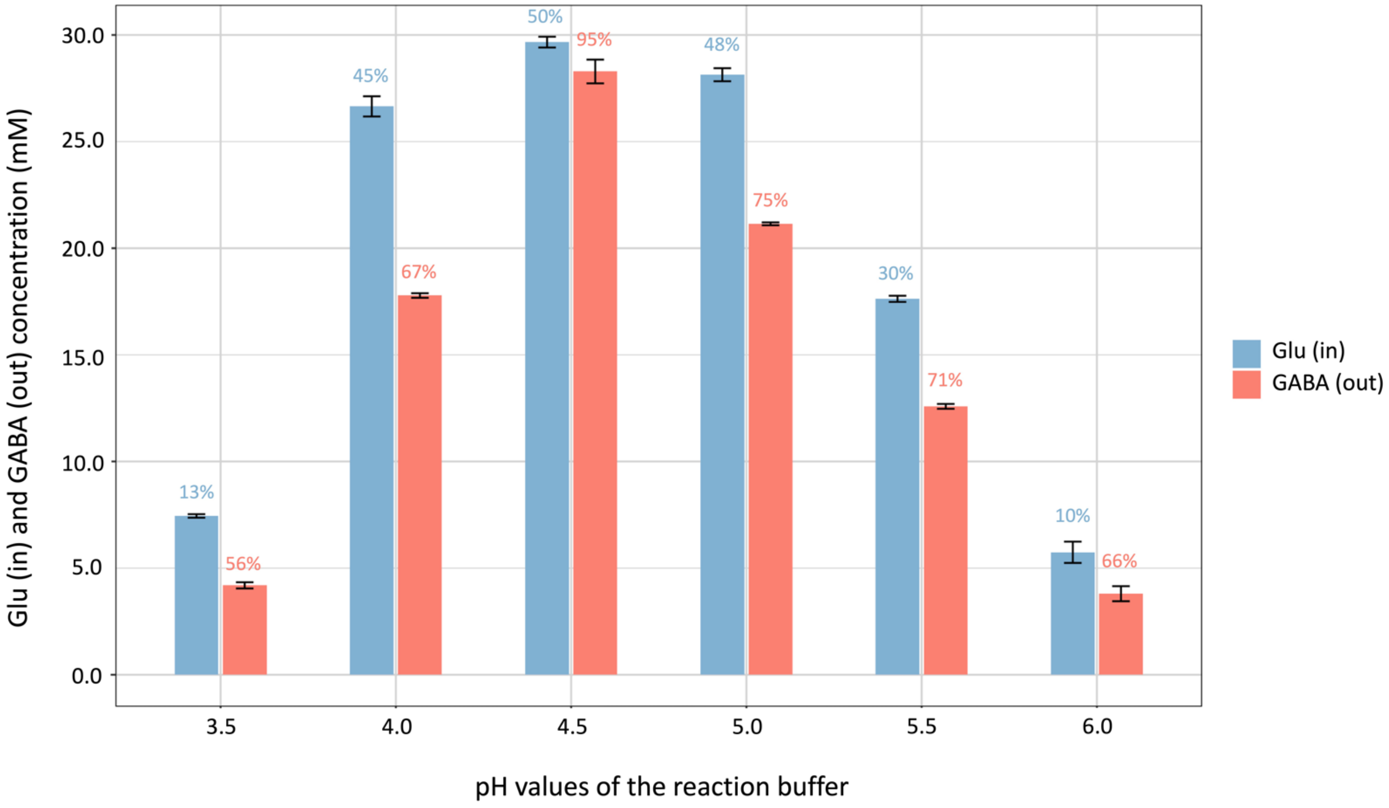

Supplementary Figure S1. Production of GABA from *Levilactobacillus brevis* LB12 in the reaction buffer (*section 2.2*). X-axis: pH values of the reaction buffer. Y-axis, blue bars: concentration (mM) of glutamate taken up by cells (Glu in) and theoretically available for bioconversion to GABA (calculated as initial Glu - residual Glu in the buffer system); Y-axis, red bars: concentration (mM) of GABA produced and extruded outside the cells (GABA out, measured in the buffer system). % on blue bars: efficiency of Glu uptake by cells; % on red bars: efficiency of Glu to GABA bioconversion. Glu and GABA concentrations, in the reaction buffer, were estimated by densitometric analysis of TLC spots.

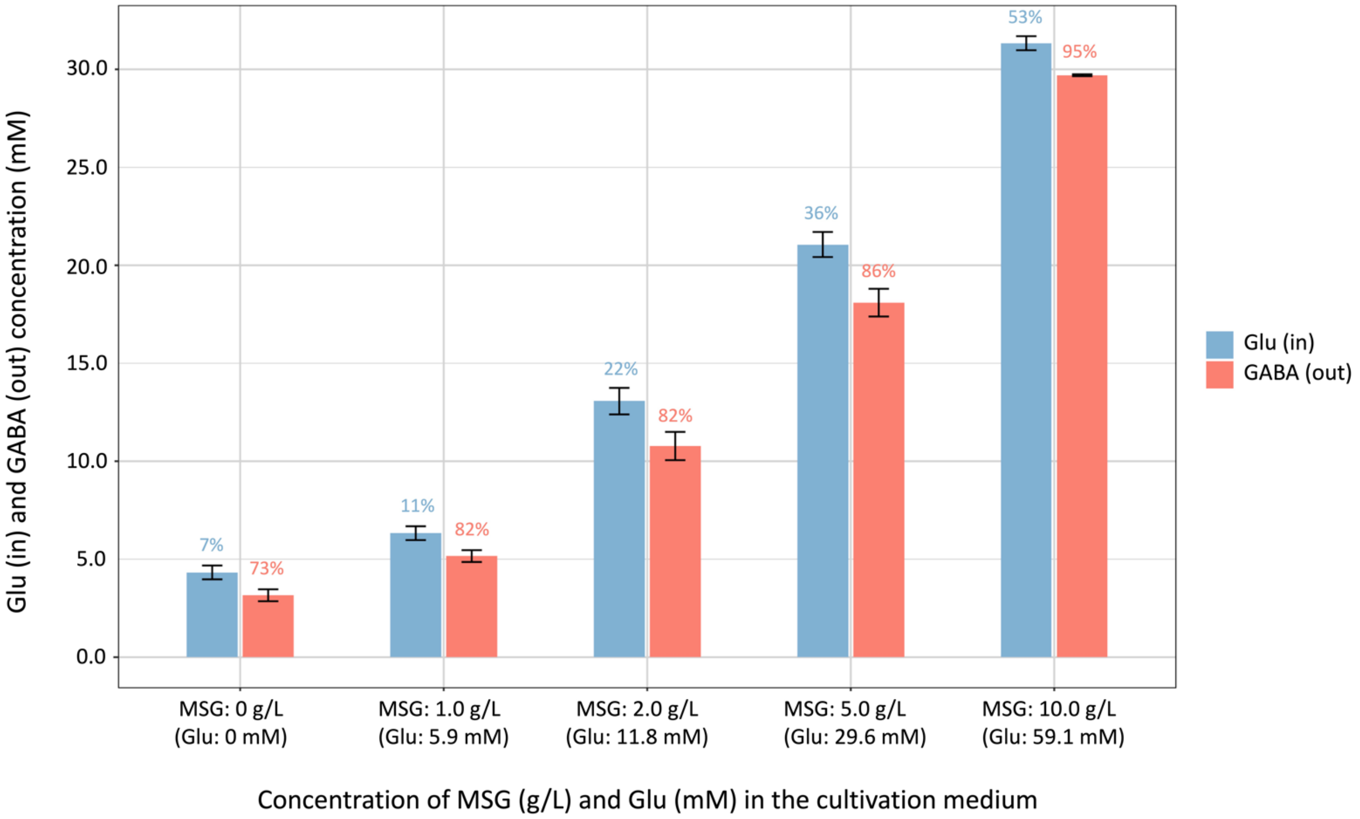

Supplementary Figure S2. Production of GABA from *Levilactobacillus brevis* LB12 cultivated in mWMB supplemented with different glutamate concentrations (*section 2.3*). X-axis: concentration of monosodium glutamate (MSG, g/L) added to mWMB; values in brackets (): final glutamate concentration (Glu, mM) in mWMB. Y-axis, blue bars: concentration (mM) of glutamate taken up by cells (Glu in) and theoretically available for bioconversion to GABA (calculated as initial Glu - residual Glu in the buffer system); Y-axis, red bars: concentration (mM) of GABA produced and extruded outside the cells (GABA out, measured in the buffer system). % on blue bars: efficiency of Glu uptake by cells; % on red bars: efficiency of Glu to GABA bioconversion. Glu and GABA concentrations, in the reaction buffer, were estimated by densitometric analysis of TLC spots.
